# Study on the Macro-Micro Mechanical Properties of Grout Consolidated Coal under Different Loading Rates

**DOI:** 10.3390/ma15248913

**Published:** 2022-12-13

**Authors:** Hongyu Pan, Junyan Wang, Guanyi Du, Kang Wang, Lei Zhang, Suinan He, Shuang Song

**Affiliations:** College of Safety Science and Engineering, Xi’an University of Science and Technology, Xi’an 710054, China

**Keywords:** grout, loading rate, consolidation body, macro-micro mechanical characteristics, the compressive strength

## Abstract

The bore hole is sealed from a sealing hole: the surrounding coal fracture permeability and grout cementation form a new consolidated body and coal material. In this paper, the characteristics of the macroscopic compressive strength, microscopic interface bending, porosity, and fractal dimension of the consolidated body were studied, and the structure strength relationship between loading rates, porosity, fractal dimension, and uniaxial compressive strength (UCS) was established. The results show that the loading rates had a great and consistent effect on the macro- and micro-mechanical properties of the consolidated body. Macroscopically, in the range of 0.1~0.4 mm/min, the UCS and elastic modulus of the solidified body increased with the increase in the loading rate, and there was a critical loading rate (*η* = 0.4 mm/min). At the microscale, with the increase in loading rates, the interface bending phenomenon, porosity, fractal dimension, and UCS of the grout and coal were consistent, showing a trend of increasing first and then decreasing. The fractal dimension was linearly correlated with the UCS and porosity. The loading rates, porosity, fractal dimension, and UCS had a multivariate nonlinear regression distribution.

## 1. Introduction

Under the influence of an external load, cracks, deformation, and instability occur in the coal and rock mass in the sealing section of a drilling hole [1,2,3], which seriously affects the quality of the sealing hole [4,5]. When drilling and sealing holes, grouting reinforcement technology is mostly used for treatment [6]. The sealing slurry penetrates and diffuses along the cracks around the hole, and cements within the coal to form a consolidated body [7,8,9]. This structure can enhance the compressive strength of the coal body around the hole and improve the sealing effect [10,11]. With different advancing speeds of the working face, different loading rates arise in the front coal, and the consolidated body exhibits different mechanical properties under different loading rates, which significantly affects the sealing effect [12,13,14]. Therefore, this effect is of great significance to guide the design of the compressive strength of consolidated bodies under the influence of the site loading rate, enhance the quality of drilling and sealing holes, and improve the efficiency of gas extraction.

At present, various scholars have conducted studies on the macroscopic mechanical strength of such consolidated bodies. Deng et al. [15] pointed out that grouting improves the cementation degree of materials. Niu et al. [16] found that the compressive strength of a material was improved after grouting. Song et al. [17] obtained the change rule that the peak strength of rock also increases with an increasing loading rate. Meanwhile, some scholars studied the change in cracks during the loading process. Le et al. [18] studied the generation form and change in cracks under loading and crack closure was found, indicating that the grouting effectively filled the cracks and reduced the stress concentration in the cracked specimens. Su et al. [19] mentioned that with the increase in loading rate, the number of cracks also increased, and the degree of rock failure increased.

In addition, research has previous been conducted on consolidation bodies from the microscopic perspective. Zhang et al. [20] discussed the pore structure change rule of CF material during expansion, and showed that the expansion agent will promote the pore development of the material, while the increase in the amount of expansion agent will increase the bearing stress of the material. Chao et al. [21] analyzed the pore structure characteristics of the cemented paste backfill samples, and the results showed that the maintenance time affected the pore structure of the specimens, and that a longer maintenance time could reduce the pore development of the specimens. Zhang et al. [22] suggested that the nature of the injection medium had little influence on the grout–coal interface. In recent years, scholars have begun to use fractal dimensions to characterize pore structures and cracks. Guo et al. [23] studied the pore characteristics of coal samples with broken particles based on fractal theory, showing that the larger the fractal dimension, the more complex the pore structure, and fractal theory can characterize the pore characteristics of coal samples. Li et al. [24] found that the fractal dimension decreased with an increase in the impact load. Bing et al. [25] suggested that with the extension in loading time, the rate of increase in the fractal dimension decreased, and the damage caused by the impact load decreased, that is, the fractal dimension can represent the degree of rock damage. Gao et al. [26] analyzed the box dimension method and found that in the crack growth stage, the fractal dimension increased rapidly with loading, while in the crack stability stage, the fractal dimension increased slowly. As the fractal dimension was introduced into the study of the microstructure of the coal and rock masses, some scholars established relationships between the fractal dimension and several macroscopic behaviors of the rock and coal samples. Wang et al. [27] established the relationship between the sample distribution characteristics and dissipated energy under impact loading. Ma et al. [28] discussed the relationship between fractal dimension and compressive strength and found that the compressive strength of the material increased as the fractal dimension increased.

In conclusion, the previous studies laid the foundation for understanding the strength variation and pore structure characteristics of consolidated bodies after grouting. However, there have been few studies on the influence of the loading rate, and the grout–coal structural plane after grouting and consolidation is rarely taken into account at the microscopic level, so there are still deficiencies in the microstructure characterization of the consolidated body and how it is related to the macroscopic properties. Therefore, this paper used a new type of liquid injected sealing material that we developed to study the macro/micro mechanical characteristics of a consolidated body with coal under different loading rates, which has a very important role in improving the quality of drilling hole sealing.

## 2. Materials and Methods

### 2.1. Preparation of Briquette and Consolidated Body Samples

**Figure 1:**
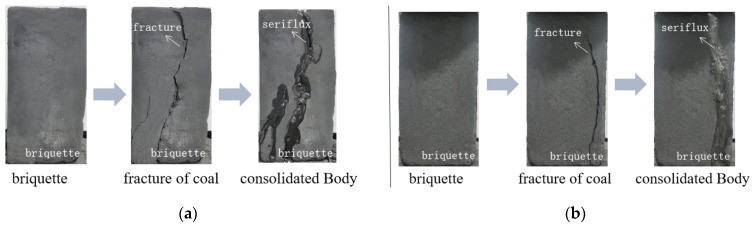
Sample preparation. (**a**) The first group; (**b**) the second group.

A briquette made of pulverized coal and cement was used simulate the properties of underground coal and rock, and its mechanical properties were basically consistent with those of coal and rock. The mass ratio of pulverized coal to cement was 3:2.5, and the samples were prepared by mixing the dry components with water. After mixing evenly, the samples were poured into a steel mold produced especially for this study. After 24 h, the mold was removed and the sample was placed in a test chamber with a constant temperature and humidity for 2 weeks, and the temperature was set at 40 °C. Next, it was loaded on a YAW-300B automatic pressure test machine (Shandong Jianli Testing Technology Co., Ltd., Jinan, China, and the test was stopped when the sample was damaged. The injector was used to inject into the damaged sample. After the slurry was completely consolidated, the consolidated body sample was prepared, as shown in Figure 1.

### 2.2. Experimental Procedure

#### 2.2.1. Determination of the Loading Rates

The different advancing speeds of the working face were manifested as different loading rates in the laboratory. The advance speed of coal mining faces in China is generally 2~5 m/d, and can reach 8 m/d in good geological conditions. According to the calculation formula between the field advance speed and the loading rate [29], the displacement loading method was adopted in this experimentation, and the loading rates ranged from 0.05 to 0.5 mm/min. Five intermediate grades were selected for the study: 0.1, 0.2, 0.3, 0.4, and 0.5 mm/min loading rates.

#### 2.2.2. Material Preparation

**Figure 2:**
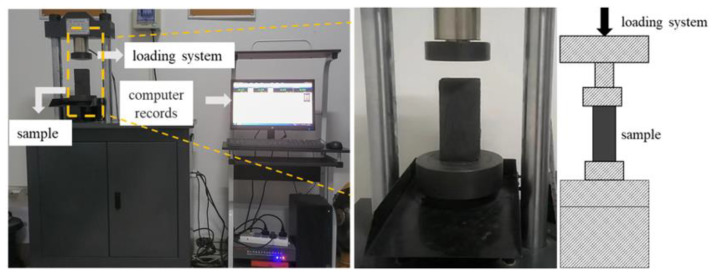
Uniaxial compression test system.

The experimental system was a single-axis compression experimental system, as shown in Figure 2. The test system adopted a YAW-300B automatic pressure testing machine, which was mainly composed of an oil source main frame, hydraulic integrated block, sensor, and PC control force measuring system. The pressure range was 0~300 kN, and the test adopted the displacement control quasi-static loading method. The axial load and axial displacement were automatically collected and recorded during the test.

Combined with the field stress analysis, the loading was stopped when the briquette sample crack was not yet completely destroyed. The surface crack of the fractured sample was filled through injection with a syringe to make a consolidated body, which was then loaded on the YAW-300B automatic pressure test machine at different rates, and the stress–strain curve and strength of the consolidated body under loading rates were obtained.

To analyze the development degree and distribution of pores and cracks in the briquette samples before and after loading, samples before and after each loading rate were cut into 5 × 5 × 5 mm^3^ standard samples for gold injection treatment, and their microstructure was observed under a Quanta 450 & IE250X-Max50 scanning electron microscope (SEM) (Beijing Yuanhaiwei Technology Co., Ltd., Beijing, China) with a magnification ratio of 6–1,000,000. For comparative analysis, the grout–coal interface part of the consolidated body samples under loading rates was observed under SEM, and SEM images of the consolidated body were obtained.

## 3. Analysis of Macroscopic Mechanical Properties under Different Loading Rates

### 3.1. Effect of Loading Rates on Stress–Strain Curve

**Figure 3:**
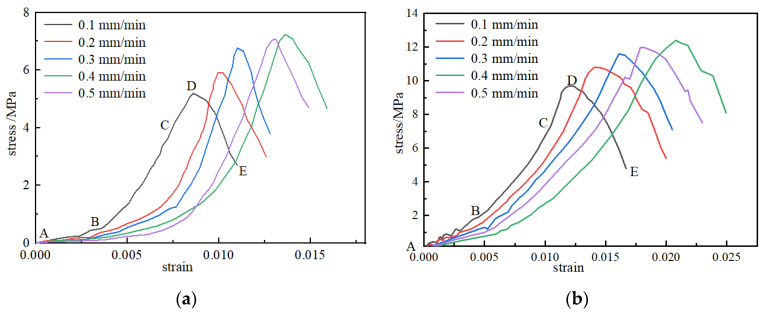
Stress–strain curves of the briquette and consolidated body under different loading rates. (**a**) Stress–strain curve of the briquette; (**b**) stress–strain curve of the consolidated body.

The stress–strain curves of the briquette and consolidation samples under different loading rates are shown in Figure 3. The stress–strain curves of the briquette and consolidated samples showed similar trends of change. Microdefects such as micropores and microcracks occur in briquettes, and the appearance of the compaction stage in the figure also confirms the existence of such microdefects. It can be seen from the figure that the stress borne by the consolidated body in the compaction stage (AB) was higher than that of the briquette sample, but the stress–strain curve of the briquette in the compaction stage was relatively slow, and the range of the elastic stage (BC) was shorter than that of the consolidated body. Because the internal pores and cracks of the sample were compressed during the compaction stage, resulting in slow deformation, some of the internal pores and cracks of the consolidated body were squeezed during the initial loading, and the volume of the pores and cracks that could be squeezed was reduced after grouting reloading, the briquette compaction stage was relatively slow. After grouting, the grout filled the holes and cracks, cemented the coal body, improved the cementation degree of the sample, and enhanced the strength of the sample. Under loading, the elastic deformation increases slowly, so the elastic stage is long. In the pre-peak failure stage (CD), the sample gradually reached the stress bearing limit under the continuous action of stress. At this stage, the sample slowly reached the stress peak and gradually exhibited macroscopic failure. In the post-peak failure stage (DE), when the stress of the sample reached the peak value, the stress decreased with loading, and the failure of the sample intensified. However, the stress of the consolidated body decreased relatively slowly compared to that of the briquette sample, which means that failure occurred slowly. The reasons for this trend are that grouting improves the integrity of the sample, the slurry and coal are cemented together, and the cementation ability of the sample is enhanced. As the loading continued, cracks caused by the failure of the sample were generated slowly, which was shown as the gradual loss of bearing capacity and the gradual instability of the sample.

When the loading rates were 0.1~0.2 mm/min, the stress adjustment phenomenon occurred in the compaction stage, and the smaller the loading rates, the more frequent the stress adjustment. The “stress adjustment phenomenon” refers to the fact that as the load continues, the specimen will continue to increase the stress until the peak stress specimen damage, and the stress adjustment phenomenon refers to the following: as the load continues due to changes in the bearing structure of the specimen, this results in a brief drop in the stress phenomenon, that is, the specimen with the loading rate of the stress appears to first increase and then decrease the characteristics of the change. The reasons for the phenomenon of “the smaller the loading rate, the more frequent the stress adjustment” are as follows. In this stage, with the loading process, the pores inside the specimen are gradually compressed, and the microcracks are extruded, expanded, and penetrated. The lower loading rate provides enough time for the internal cracks of the specimen to develop and expand in the compaction stage, which leads to the change in the bearing structure, the effective bearing area of the specimen is temporarily reduced, the stress gradually decreases, and the stress adjustment phenomenon appears. During the loading process, due to sufficient time, the internal cracks of the specimen show dynamic crack closure and development, which makes the dynamic adjustment of the bearing structure take place, and the phenomenon of multiple stress adjustment occurs as the stress adjustment is frequent.

At loading rates of 0.3~0.4 mm/min, the stress adjustment phenomenon occurred in the elastic stage of the stress–strain curve. At this stage, new cracks are gradually generated, the particles with weak internal connections are destroyed, and part of the energy is absorbed, so the stress decreases and stress adjustment occurs.

When the loading rates were 0.5 mm/min, the stress adjustment phenomenon occurred in the post-peak stage of the stress–strain curve. Due to the self-reconstruction and adjustment of the structure after crack failure, the effective bearing area of the sample will increase temporarily, so the stress will increase temporarily, and the stress adjustment phenomenon occurs.

The briquette sample also underwent a stress adjustment phenomenon, but was weaker than that of the consolidated body. Due to the low strength and strong brittleness of the briquettes, the deformation, fracture, and reconstruction phenomenon of the internal particles was weaker, the effective bearing area of internal holes and cracks changed less, and the stress adjustment phenomenon was weaker.

### 3.2. Effect of Loading Rates on Mechanical Properties

#### 3.2.1. Effect of Loading Rates on UCS

Table 1 shows the UCS and standard deviations of the of briquette and consolidated body. The average UCS of the briquette was 6.46 MPa, and the average UCS of the consolidated body was 11.3 MPa. After grouting reinforcement, the strength increased by 74.9%. The standard deviation of the measured compressive strength of the briquette and consolidated body was calculated to be in the range of 0.2~0.4 MPa, indicating that the strength of the test data fluctuated little, the deviation was small and the test results were stable.

**Figure 4:**
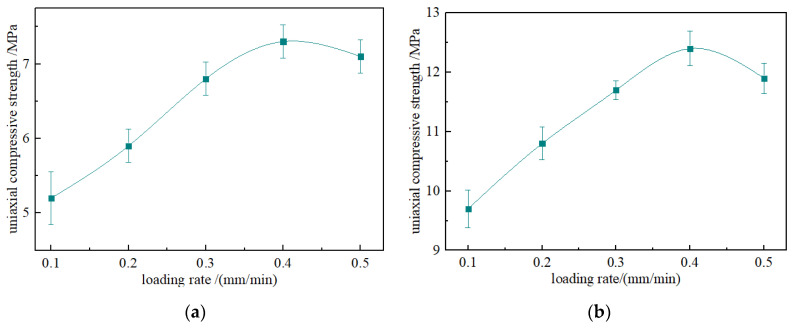
The compressive strength of the briquette and consolidated body under different loading rates. (**a**) Compressive strength of briquette; (**b**) compressive strength of consolidated body.

Based on the compression stress–strain curve of the consolidated body under compression, the average elastic modulus of the consolidated body was calculated according to Equation (1), the mechanical parameters of the consolidation were calculated, and the mechanical parameters were plotted as shown in Figure 4.

(1) $$E=\frac{\sigma_{b}-\sigma_{a}}{\varepsilon_{b1}-\varepsilon_{a1}}$$

where *a* and *b* are the start and end stresses of the online elastic stage of the stress–strain curve. *a*1 and *b*1 are the corresponding strain values of *a* and *b*, respectively.

It can be seen from the figure that there was no linear relationship between the UCS of the briquette and consolidated body and the loading rates. There is a “critical loading rate” between the strength and the loading rates, which is the rate when the compressive strength peaks. It was found that the relationship between the loading rates and UCS is a Gaussian distribution, and the fitting relationships expressed in Equations (2) and (3) characterize the relationships between the UCS of the briquette and consolidated body with the loading rates.

(2) $$\sigma_{c}{}_{a}=4.63824+2.69289e^{-\frac{{\left(\eta -0.42238\right)}^{2}}{0.066}}$$

(3) $$\sigma_{c}{}_{b}=8.73043+3.59525e^{-\frac{{\left(\eta -0.40727\right)}^{2}}{0.07314}}$$

where *σ_c_* is the compressive strength of the sample in MPa; *η* is the loading rate in mm/min.

With the increase in loading rate, the increase in the compressive strength of the consolidated body decreases, and the compressive strength decreases when the critical loading rate is reached. When the loading rate was 0.1~0.2 mm/min, the increase in compressive strength was 11.3%, and when the loading rate was 0.3~0.4 mm/min, the increase was only 5.9%. The reasons are as follows: when the loading rate is low, the pores and cracks in the consolidated body continue to develop, and before the stress peak, the pores and cracks are fully developed, which consumes more energy, leading to the reduction in the compressive strength of the consolidated body. When the loading rate is high, the development and expansion time of the internal pores and cracks of the consolidated body is insufficient, the internal integrity is relatively high, and the stored energy is greater, so the compressive strength also increases. The loading rate increases gradually, the increase in stress decreases, and the sample gradually reaches the upper limit of the bearing capacity, so the rate of increase slows. The strength decreases after reaching the critical loading rate, which can be expressed by introducing the applied load, *σ_1_*, in the compression process of the sample [30].

(4) $$\sigma_{1}=E\frac{\eta t}{L}A_{r}$$

where *E* is the elastic modulus; *η* is the loading rate; *t* is the loading time; *L* is the axial length of the sample; *A_r_* is the effective bearing area.

With the increase in the loading rate, the damage area of some bearing structures increased and the bearing capacity decreased due to the existence of internal pores and cracks in the sample, which easily formed stress concentrations and reached the bearing limit in advance, resulting in the failure of a large number of internal bearing structures.

#### 3.2.2. Effect of Loading Rates on Elastic Modulus

**Figure 5:**
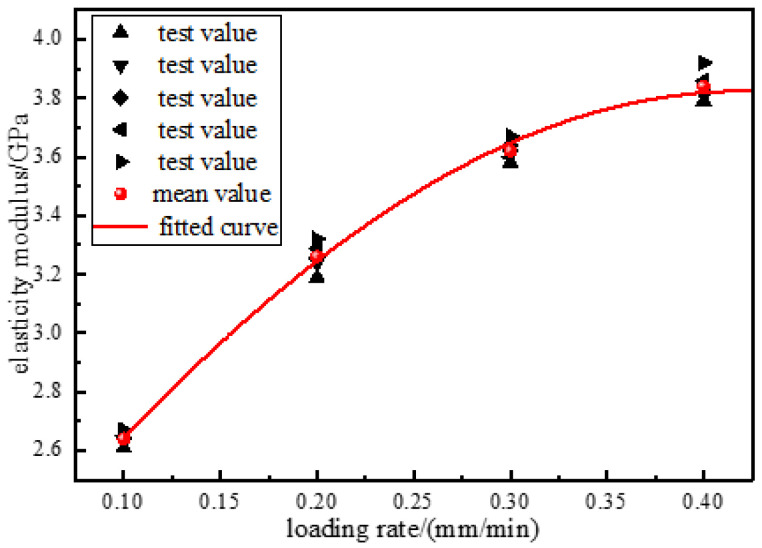
Elastic modulus of the consolidated body.

According to Figure 5, the elastic modulus of the consolidated body first increased and then decreased with the loading rate, and the distribution of the elastic modulus and loading rate was found to be Gaussian function. When the loading rate was 0.1~0.2 mm/min, the increase in the elastic modulus reached 23.5%, and when the loading rate was 0.3~0.4 mm/min, the increase decreased to 6%, and the rate of increase first increased and then decreased. This has a similar change law with the change in the compressive strength of the solidification and loading rate, that is, the distribution of the modulus of elasticity and loading rate is a Gaussian function distribution; the reason for this is similar to the previous analysis of the loading rate with the loading rate, specifically: under the load, the internal fissures of the specimen showed extrusion closure and the accumulation of elastic energy. As the loading rate increases, the internal pores and fissures of the consolidation body cannot be fully extended, the internal integrity is high, and the internal structure has a strong ability to resist deformation, so the elastic modulus increases. After reaching the critical rate, due to the existence of internal pores and cracks in the specimen, the damaged area of part of the bearing structure increases, the internal integrity decreases, the bearing capacity decreases, the stress concentration is easily formed, the bearing limit is reached in advance, leading to the destruction of a large number of internal bearing structures, and the ability of the specimen to resist deformation decreases, so the elastic modulus decreases. Its overall change trend is consistent with the change in compressive strength, which is also consistent with the relationship between the loading rate and compressive strength obtained in earlier research by other authors [31].

**Figure 6:**
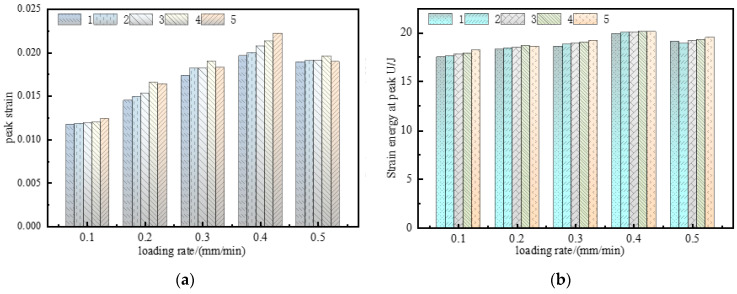
Mechanical parameters of consolidation at different loading rates. (**a**) Peak strain of consolidated body; (**b**) strain energy at the peak of the consolidated body.

In Figure 6, with increasing loading rates, both the peak strain and elastic strain energy at the peak first increased and soon decreased. With the increase in loading rates, the internal microfractures did not fully developed, and the integrity was high. The work undertaken by the load is mainly stored in the form of deformation energy, and the dissipative energy generated by friction and extrusion between fractures is small, so the elastic strain energy at the peak is high and the strain increases. When the critical loading rate is reached, the strength of the specimen decreases, the energy consumed by the defect crack propagation decreases, the accumulated elastic energy decreases, and the strain decreases.

### 3.3. Effect of Loading Rates on Failure Characteristics

**Figure 7:**
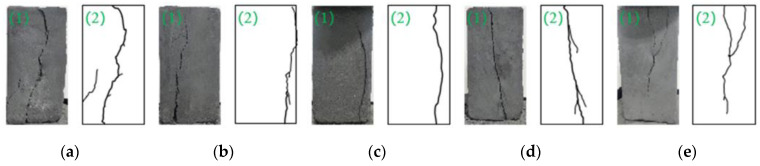
Failure types of the briquette under different loading rates. (**a**) *η*_7−*a*_ = 0.1 mm/min; (**b**) *η*_7−*b*_ = 0.2 mm/min; (**c**) *η*_7−*c*_ = 0.3 mm/min; (**d**) *η*_7−*d*_ = 0.4 mm/min; (**e**) *η*_7−*e*_ = 0.5 mm/min.

Figure 7 is a sketch of the surface failure characteristics of the briquette and consolidated body. The diagram shows that the loading rates have a great impact on the failure form of the samples. The briquette samples mainly produce cracks along the axial direction, among which, the main cracks of samples A~D mostly occurred along the upper part and ran through the bottom, accompanied by small secondary cracks. As the loading rates increased, the fracture degree of the sample increased, more new cracks formed, and the number of microcracks and secondary cracks increased. There were two main cracks in the upper end of the sample, which gradually converged into one and penetrated to the bottom, and the secondary cracks were reduced. Due to the load on the upper end of the sample, the stress concentration in the weak part of the local bond increased, and two main cracks were formed. Moreover, the bearing capacity of the sample itself is limited. When the critical loading rate is reached, the strength decreases, and the degree of fracture decreases including that of secondary cracks.

**Figure 8:**
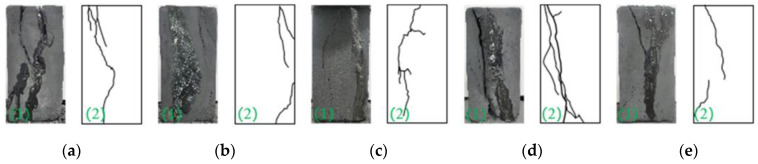
Failure types of the consolidated body under different loading rates. (**a**) *η*_8−*a*_ = 0.1 mm/min; (**b**) *η*_8−*b*_ = 0.2 mm/min; (**c**) *η*_8−*c*_ = 0.3 mm/min; (**d**) *η*_8−*d*_ = 0.4 mm/min; (**e**) *η*_8−*e*_ = 0.5 mm/min.

As shown in Figure 8, a crack in the consolidated body sample first occurred in the ungrouted area, and the reasons are as follows: Slurry infiltration into the fracture causes fissure filling before the weak positions bond together, which improves the internal integrity and strength of the specimen. Therefore, due to the load and grouting area, grouting can effectively improve the crack inducing stress concentration and intensity, and improve the grouting effect and thus the sample integrity.

Grouting can improve the bearing capacity of the sample, enhance the degree of cementation, increase the loading rates, and first increase and then decrease the crack complexity, that is, the crack undergoes an evolution from simple to complex to simple. When the loading rate is low, the internal original crack friction results in extrusion, the strength consumption is large, and a few new cracks are formed. With the increase in loading rates, the extrusion time of internal microcracks is short, and the stress is not fully dispersed. A stress concentration easily forms at the crack, and new cracks form. When the critical loading rates are reached, the strength of the sample decreases, the bearing capacity decreases, and the crack generation decreases.

## 4. Analysis of Micromechanical Properties under Different Loading Rates

### 4.1. Pore Structure Characteristics with Loading Rates

To investigate the microscopic characteristics of briquettes and the consolidated body before and after loading, scanning electron microscopy experiments were conducted on the specimens before and after loading at each rate to obtain the microstructure of the specimens.

**Figure 9:**
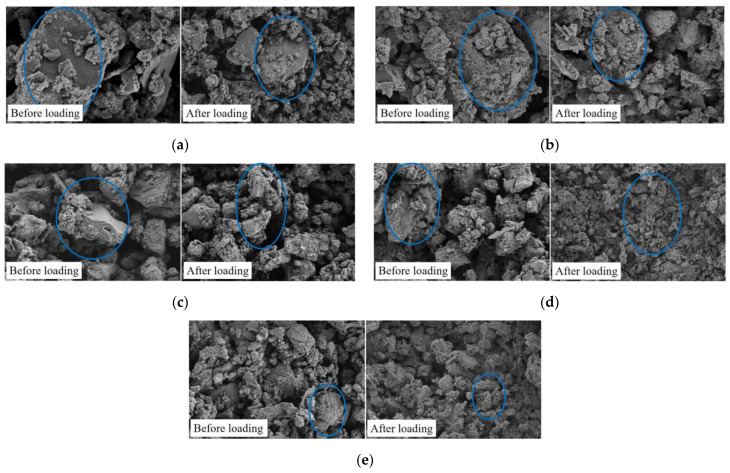
Pore structure of the briquette under different loading rates. (**a**) *η*_9−*a*_ = 0.1 mm/min; (**b**) *η*_9−*b*_ = 0.2 mm/min; (**c**) *η*_9−*c*_ = 0.3 mm/min; (**d**) *η*_9−*d*_ = 0.4 mm/min; (**e**) *η*_9−*e*_ = 0.5 mm/min.

**Figure 10:**
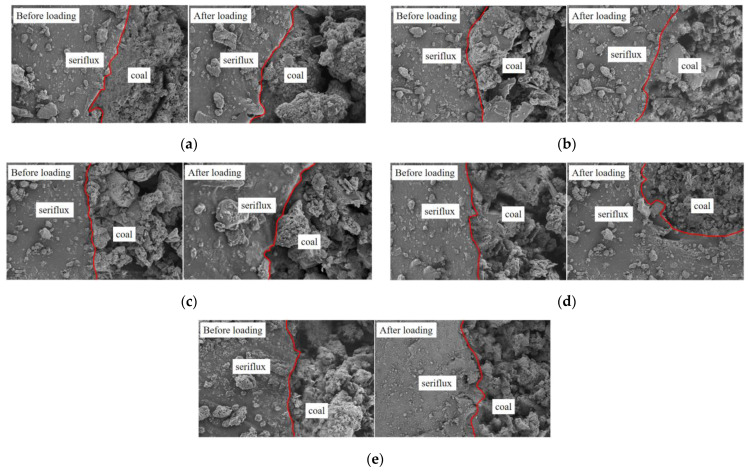
SEM images of the interface junction of new grout and coal before and after loading. (**a**) *η*_10−*a*_ = 0.1 mm/min; (**b**) *η*_10−*b*_ = 0.2 mm/min; (**c**) *η*_10−*c*_ = 0.3 mm/min; (**d**) *η*_10−*d*_ = 0.4 mm/min; (**e**) *η*_10−*e*_ = 0.5 mm/min.

The microstructure of the briquette samples with a magnification of 2000 times is shown in Figure 9, and Figure 10 shows the SEM images at the grout–coal interface before and after loading. Figure 9 shows that the loading rates have a great impact on the pore structure of the briquette samples. As the loading rate increases, the small particles in the samples increase first and then decrease. At the loading rates of (a) and (b), the loading rates were low, the fracture degree was small, and the fracture occurred in a large particle. Due to the low loading rates, the time to reach the peak stress was long, so there was enough time for particle reconstruction and breakage, and the deformation remained small. Therefore, lower loading rates often lead to larger particles and a lower degree of specimen fracture. The loading rates of (c) and (d) were higher, and the small particles in the sample increased in number. Due to the high loading rates, the sample absorbed more energy before fracture, and the time to reach the peak stress was shorter, so there was not enough time for particle reconstruction, and the pores were not fully developed. The deformation was large and more fractures occurred during loading, so the number of small particles gradually increased. (e) When the specimen reached the critical loading rates, due to the limitation of its bearing capacity, the crack at the defect was activated and transmitted part of the stress concentration, so the bearing capacity decreased and the degree of fracture decreased.

In Figure 10, the interface of the consolidation slurry and coal body was not level or smooth, and coal slurry particles staggered, in addition, the new type of slurry coal interface was relatively fuzzy, with no obvious interface phenomenon. The grout could infiltrate into the coal pores, the coal matrix further bonded together, the interfacial compatibility was good, the bonding force was strong, and the coal strength was improved. The reasons for these outcomes are as follows: the viscosity of the new grout material improved the bonding force between the grout and coal, re-cemented the coal body containing cracks into a whole, and delayed volume shrinkage until after the new grout solidified, avoiding the defects caused by volume shrinkage and grout separation from the coal matrix, thus enhancing the strength of the consolidated body.

The grout–coal interface changed little under loading. Because the new hole sealing slurry is an elastomer, after consolidation, the density of bubbles was high, the change under pressure was small, the stability was good, and the strength was high. In addition, under different loading rates, the interface was subjected to different degrees of compressive failure, resulting in aggregates and a minor degree of bending failure phenomenon. With increasing loading rates, the interface bending degree first increased and then decreased. Due to the small loading rates, the internal particles underwent slow extrusion deformation, the degree of deformation was small, and the degree of bending at the interface was small. The larger the loading rates, the faster the extrusion deformation rate of the internal particles, the larger the deformation degree, and the greater the bending degree of the interface. After the critical loading rates was reached, some bearing structures reached the bearing limit due to the internal defects of the sample, which led to a decrease in the bearing capacity and the degree of deformation.

### 4.2. Porosity Evolution with Loading Rates

**Figure 11:**
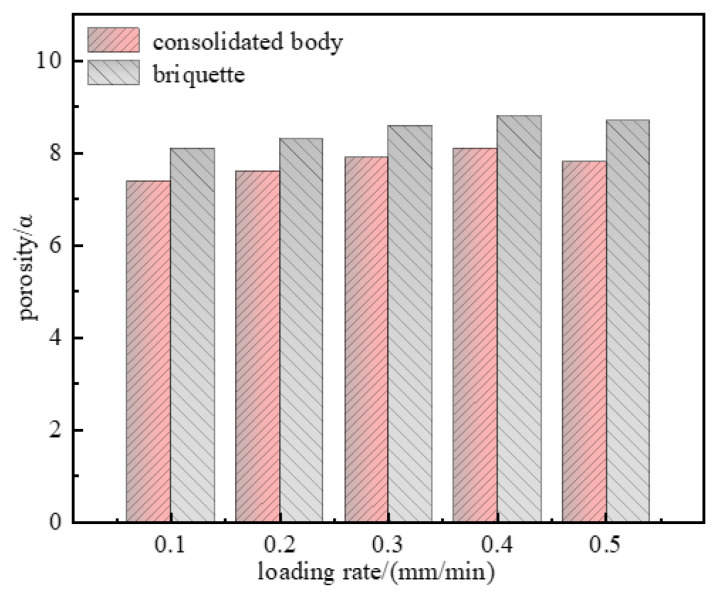
Porosity of the briquette and consolidated body under different loading rates.

To quantitatively analyze the changes in the internal pore structures of the consolidated body and briquette and characterize their microscopic characteristics, the porosity was calculated based on their SEM images, and the relationship between the loading rates and porosity was drawn, as shown in Figure 11. Clearly, the porosity of the briquette was higher than that of the consolidated body, indicating that grouting can improve the integrity of the fractured coal body and reduce the degree of specimen breakage. The porosity first increased and then decreased with the increasing loading rates. The larger the loading rates, the larger the amount of energy stored in the specimen due to deformation, the larger the change in porosity during failure, the higher the degree of fracture, and the higher the porosity. After the critical loading rates, some defects were activated and absorbed part of the energy, which led to a decrease in the pore deformation, fracture degree, and porosity, which is consistent with the change in pore structure under different loading rates. The reason is that after reaching the critical loading rate, some microcracks and pores inside the specimen were activated to extend and penetrate to share part of the stress concentration, and the cracks were activated while absorbing part of the energy, which reduced the energy required for specimen damage, and the microcracks could not be fully developed, the damage inside the specimen was reduced, and the pore deformation was reduced.

### 4.3. Fractal Dimension Evolution with Loading Rates

To explore the fractal characteristics of the briquette and consolidated body under the loading rates, the box dimension method was used to count the FD (*D*). The results of the FD of the briquette and consolidated body fractures are shown in Table 2.

**Figure 12:**
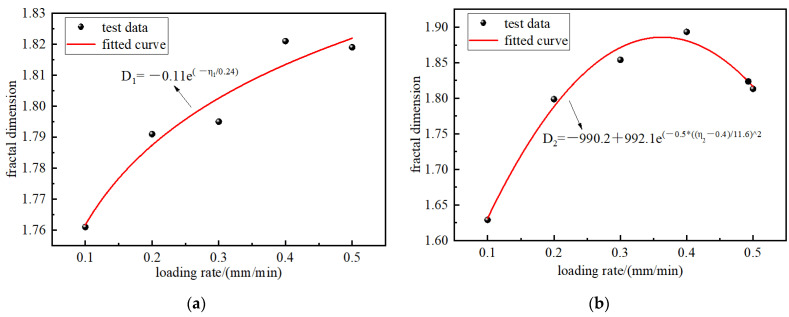
Relationship between the FD and loading rates. (**a**) Briquette; (**b**) consolidation.

As seen from Table 2, the increase in loading rates increased the FD of the briquette and consolidated body fractures. To understand the relationship between the loading rates and FD more clearly, the relationship between the FD and loading rates is shown in Figure 12.

According to Figure 12, the loading rates had a certain impact on the FD. The fitting results indicate that the FD of the surface cracks of the briquettes after loading fracture changed exponentially with the loading rates. The faster the loading rates, the larger the FD, indicating that as the fracture degree increases, smaller particles are broken, and the internal cracks become more complex with the fractal characteristics of a briquette, so the FD of a consolidated body is lower. Due to the high density of bubbles and small pores of the new grout, this grout can bond well with coal, fill some pores, reduce the existence of cracks, improve the strength of the consolidated body, and reduce the damage degree of the sample, which will reduce the FD.

The FD and loading rates of the surface cracks of the consolidated body showed a Gaussian function distribution, and the complexity of the fragments increased first and then decreased. Below 0.5 mm/min, the faster the loading rates, the larger the FD, the more complex the pore structure, the more energy consumed, and the higher the damage degree. At 0.5 mm/min, the FD decreased. As the larger loading rates activated the defect cracks inside the sample and transmitted part of the stress concentration, the damage degree decreased, the fracture complexity decreased, and the FD decreased, which is consistent with the change law of the UCS with the loading rates.

## 5. Structure–Strength Relationship between Loading Rates, Porosity, FD, and UCS

According to previous studies and the above analysis, there is a certain relationship between the compressive strength of samples and the internal pore structure [32,33,34]. The relationship between pore characteristics and the mechanical properties of the samples helps us better understand the material properties from the microscale and macroscale perspectives. Therefore, the relationship between the pore characteristics and the compressive strength of the briquette and consolidated body materials was analyzed.

**Figure 13:**
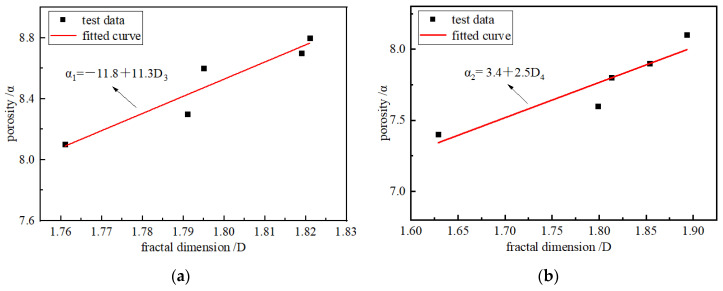
FD and porosity. (**a**) Briquette; (**b**) consolidated body.

Figure 12 shows that as the loading rates increased, the FD increased first and then, according to the previous analysis, the porosity increased with the loading rates and ultimately decreased; this shows that there is a specific concern between the FD and porosity. To explore the concern between FD and pore structure, the porosity and FD relation curve is given in Figure 13, which shows the fitting. There was a linear correlation between the FD and porosity of the briquette and consolidated body.

The samples show that the greater the internal pore porosity, the more complex the internal structure. The graph shows that the good correlation with porosity and FD increased with the increases in FD, therefore, the FD of the sample can be characterized by the internal pore structure characteristics, reflecting its microscopic characteristics.

**Figure 14:**
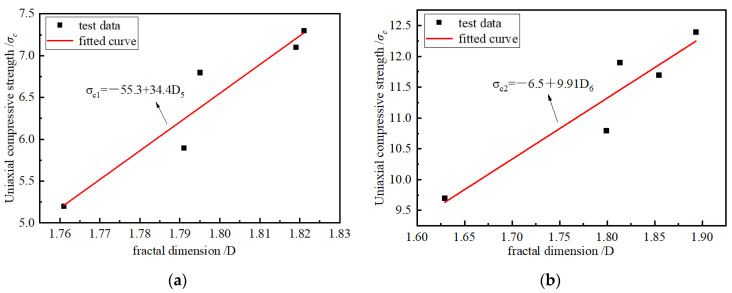
FD and UCS. (**a**) Briquette; (**b**) consolidated body.

According to Figure 5 and Figure 12, with an increase in the loading rates, the UCS of the briquette and consolidated body had extreme values. The larger the UCS, the more cracks are produced in the sample, the larger the degree of fracture, the larger the proportion of small particles, and the larger the FD. The FD can further explain the sample internal pore fissure structure. To further explore the influence of the UCS of the specimen pore distribution, research on the coal and UCS of the consolidated body and the relationship between the FD and UCS of the drawing and the relationship between the FD curve are as shown in Figure 14. The fitting and FD increase as the UCS increases, reflecting a more complex internal crack structure, and the relationship between the UCS and FD is a linear function. Clearly, the larger the UCS of the briquette or consolidated body, the more complex the pore structure, and the more closely the macroscopic phenomenon corresponds to the microscopic phenomenon.

**Figure 15:**
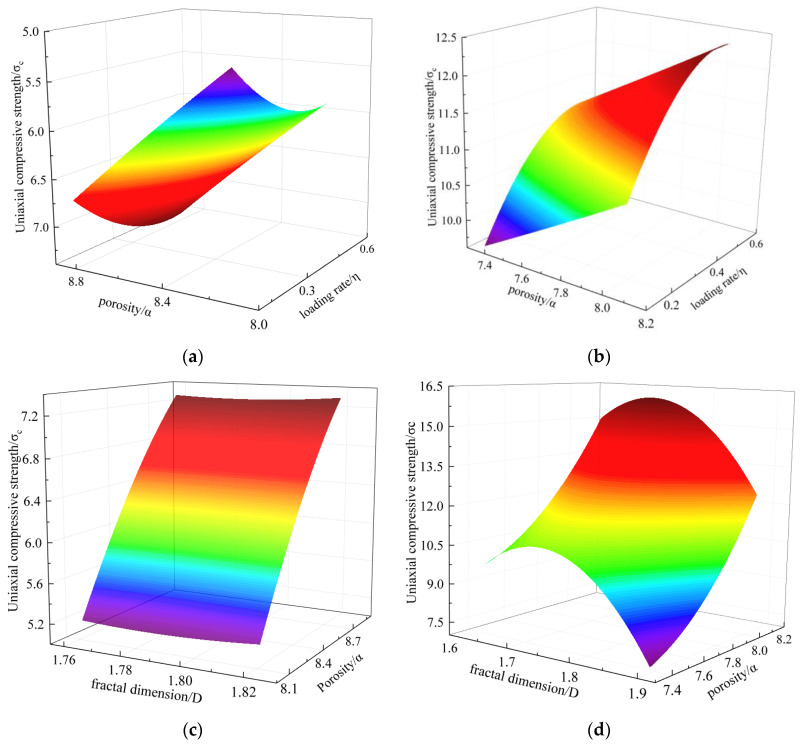
Surface diagram of the briquette and consolidated body fitting. (**a**) Porosity, loading rates, and UCS of the briquette; (**b**) Porosity, loading rates, and UCS of the consolidated body; (**c**) Porosity, FD, and UCS of the briquette; (**d**) Porosity, FD, and UCS of the consolidated body.

Through the above analysis, we can see that the FD and porosity have a linear relationship. The UCS and loading rates had a Gaussian distribution, as did the loading rates, porosity, and FD. To further explore the relationship between them, we drew the loading rates, porosity, and UCS as a 3D surface. This is shown in Figure 15, and Table 3 and Table 4 are fitting formulas.

According to the fitting formula, the briquette and consolidated body had similar functional relations; that is, the briquette and consolidated body had similar evolutions. From Table 3, the relationship between the loading rates, porosity, FD, and UCS of the briquettes can be deduced as follows:

(5) $$\sigma_{c1}=1+18.3\alpha^{2}-65.3\alpha -2.7\eta^{2}+2.4\eta -0.7D^{2}+12.8D$$

Similarly, the relationship between the loading rates, porosity, FD, and UCS of the consolidated body can be deduced from Table 4, as follows:

(6) $$\sigma_{c2}=1-60.2\alpha^{2}+207.6\alpha -5.6\eta^{2}+5.2\eta -3.4D^{2}-48.3D$$

For the above analysis and multivariate nonlinear regression, to build a model of the coal and consolidated body for study under any loading rates, porosity, and FD and the structure–compressive strength relationship, we established a model of coal and consolidated body contact that considers the macro/micro-mechanical properties.

## 6. Discussions and Conclusions

By measuring the macro/micro mechanical properties of the briquettes and consolidated bodies under loading at different rates, the changes in UCS, porosity, and FD were analyzed, and the relationship between the macro- and micro-mechanical properties was established. The following conclusions can be drawn from the current study.

(1) The loading rates, UCS, and macroscopic cracking of the briquette and consolidated body first increased and then decreased, and there were critical loading rates (*η* = 0.4 mm/min). At the critical loading rates, the UCS peaked, and the bending degree of the microscale interface of the consolidated body also peaked.
(2) The macroscopic and microscopic phenomena of the briquette and consolidated body were consistent, there was a linear relationship between the microscopic porosity and FD, and the variation in the microscopic porosity and FD was consistent with that in the macroscopic UCS under different loading rates.
(3) By analyzing the relationship among the loading rates, porosity, FD, and UCS of the briquettes and consolidated bodies, a multivariate nonlinear regression equation was obtained, and the regression effect was remarkable. The relationship can be used to guide the design of the compressive strength of a consolidated body.

The previous study showed that grouting could increase the compressive strength of the fractured coal body and improved the mechanical properties of the fractured coal body. The current study confirms that grouting improves the strength of a fractured coal body, and that the loading rate has an effect on the macro- and micro-mechanical properties of a slow-setting slurry-coal solidification body, the macro- and micro-mechanical properties change consistently at different loading rates, and there is a critical loading rate (*η* = 0.4 mm/min). Meanwhile, by analyzing the interrelationship between porosity, fractal dimension, loading rate, and compressive strength, it was concluded that the porosity, fractal dimension, loading rate, and compressive strength had a multivariate nonlinear regression distribution to construct a link between the macroscopic mechanical properties of the slow-setting slurry-coal solidification body, which is important for the design of the slurry-coal solidification body strength.

In general, this paper established the connection between the macro- and micro-mechanical properties of the cemented solids by analyzing the macro- and micro-mechanical properties of the slow-setting slurry-coal cemented solids, but it should be noted that the equation for the compressive strength of the cemented solids established in this paper is only applicable to the range of loading rates studied in this paper, and whether it is applicable to other ranges of loading rates needs to be further explored by researchers. At the same time, this paper only studied the new liquid slow-setting sealing material made by this paper and the solidification body condensed by coal, so whether it is applicable to other sealing materials needs to be studied and discussed by the researchers.

Based on the above study, the following recommendations can be made to scholars. This paper was based on the loading rate range selected in the paper to establish the solidification compressive strength equation, so that scholars can set a different loading rate range to study the connection between the macroscopic mechanical properties of solidification. At the same time, only homemade sealer materials were used in this paper, which is a single type of material. Scholars can select different sealer materials, compare the differences between them, and explore whether the solidus compressive strength equation is applicable to other types of sealer materials.

## Figures and Tables

**Table 1:** The compressive strength test results of the briquette sample and consolidated body sample.

Sample Type	Loading Rate (mm/min)	Compressive Strength/(MPa)	Standard Deviation	Sample Type	Loading Rate (mm/min)	Compressive Strength/(MPa)	Standard Deviation
Coal briquette	0.1	5.2	0.35355	consolidated body	0.1	9.7	0.31623
	0.2	5.9	0.22361		0.2	10.8	0.27386
	0.3	6.8	0.22361		0.3	11.7	0.15811
	0.4	7.3	0.22361		0.4	12.4	0.29155
	0.5	7.1	0.22361		0.5	11.9	0.25495

**Table 2:** FD of the fracture of the briquette and consolidated body under different loading rates.

Sample	*η* (mm/min)	Fitting Formula	*D*	Sample	*η* (mm/min)	Fitting Formula	*D*
briquette	0.1	*y* = 12.57 − 1.761*x*	1.761	consolidated body	0.1	*y* = 11.91 − 1.629*x*	1.629
	0.2	*y* = 12.63 − 1.791*x*	1.791		0.2	*y* = 12.53 − 1.799*x*	1.799
	0.3	*y* = 12.70 − 1.795*x*	1.795		0.3	*y* = 12.97 − 1.854*x*	1.854
	0.4	*y* = 12.87 − 1.821*x*	1.821		0.4	*y* = 12.85 − 1.893*x*	1.893
	0.5	*y* = 10.86 − 1.819*x*	1.819		0.5	*y* = 12.58 − 1.813*x*	1.813

**Table 3:** Fitting formula of the coal briquette.

Parameter	Formula	*R^2^*
Porosity loading rates and UCS	$\sigma_{c}=1+0.2\alpha^{2}-1.1\alpha -5.4\eta^{2}+4.8\eta$	0.99
Porosity FD and UCS	$\sigma_{c}=1+36.3\alpha^{2}-129.6\alpha -1.3D^{2}+25.6D$	0.99

where *α* is the porosity; *η* is the loading rate in mm/min; *D* is the fractal dimension; *σ*_c_ is the uniaxial compressive strength in MPa.

**Table 4:** Fitting formula of the consolidated body.

Parameter	Formula	*R^2^*
Porosity loading rates and UCS	$\sigma_{c}=1+0.1\alpha^{2}+0.5\alpha -11.1\eta^{2}+10.5\eta$	0.99
Porosity FD and UCS	$\sigma_{c}=1-120.5\alpha^{2}+414.7\alpha +6.7D^{2}-96.4D$	0.99

## Data Availability

All authors approved the publication of the paper. The data used to support the findings of this study are available from the corresponding author upon request.
